# Immunohistochemically detectable metallothionein expression in malignant pleural mesotheliomas is strongly associated with early failure to platin-based chemotherapy

**DOI:** 10.18632/oncotarget.24962

**Published:** 2018-04-27

**Authors:** Fabian D. Mairinger, Jan Schmeller, Sabrina Borchert, Michael Wessolly, Elena Mairinger, Jens Kollmeier, Thomas Hager, Thomas Mairinger, Daniel C. Christoph, Robert F.H. Walter, Wilfried E.E. Eberhardt, Till Plönes, Jeremias Wohlschlaeger, Bharat Jasani, Kurt Werner Schmid, Agnes Bankfalvi

**Affiliations:** ^1^ Institute of Pathology, University Hospital Essen, University of Duisburg-Essen, Essen, Germany; ^2^ Department of Pneumology, Helios Klinikum Emil von Behring, Berlin, Germany; ^3^ Department of Pathology, Helios Klinikum Emil von Behring, Berlin, Germany; ^4^ Department of Medical Oncology, West German Cancer Center, University Hospital Essen, University of Duisburg-Essen, Essen, Germany; ^5^ Department of Internistic Oncology, Kliniken Essen Mitte, Essen, Germany; ^6^ Ruhrlandklinik, West German Lung Centre, University Hospital Essen, University of Duisburg-Essen, Essen, Germany; ^7^ Department of Thoracic Surgery and Thoracical Endoscopy, Ruhrlandklinik, University Hospital Essen, University of Duisburg-Essen, Essen, Germany; ^8^ Department of Pathology, Diakonissenkrankenhaus Flensburg, Flensburg, Germany; ^9^ Department of Pathology, Targos Molecular Pathology GmbH, Kassel, Germany

**Keywords:** malignant pleural mesothelioma, metallothionein, overall survival, prognostic biomarker, platin-based chemotherapy, Pathology

## Abstract

**Background:**

Malignant pleural mesothelioma (MPM) is a biologically highly aggressive tumor arising from the pleura with a dismal prognosis. Cisplatin is the drug of choice for the treatment of MPM, and carboplatin seems to have comparable efficacy. Nevertheless, cisplatin treatment results in a response rate of merely 14% and a median survival of less than seven months. Due to their role in many cellular processes, methallothioneins (MTs) have been widely studied in various cancers. The known heavy metal detoxifying effect of MT-I and MT-II may be the reason for heavy metal drug resistance of various cancers including MPM.

**Methods:**

105 patients were retrospectively analyzed immunohistochemically for their MT expression levels. Survival analysis was done by Cox-regression, and statistical significance determined using likelihood ratio, Wald test and Score (logrank) tests.

**Results:**

Cox-regression analyses were done in a linear and logarithmic scale revealing a significant association between expression of MT and shortened overall survival (OS) in a linear (p=0.0009) and logarithmic scale (p=0.0003). Reduced progression free survival (PFS) was also observed for MT expressing tumors (linear: p=0.0134, log: p=0.0152).

**Conclusion:**

Since both, overall survival and progression-free survival are negatively correlated with detectable MT expression in MPM, our results indicate a possible resistance to platin-based chemotherapy associated with MT expression upregulation, found exclusively in progressive MPM samples. Initial cell culture studies suggest promoter DNA hypomethylation and expression of miRNA-566 a direct regulator of copper transporter SLC31A1 and a putative regulator of MT1A and MT2A gene expression, to be responsible for the drug resistance.

## INTRODUCTION

Malignant pleural mesothelioma (MPM) is a predominantly asbestos-related and biologically highly aggressive tumor associated with a remarkably poor prognosis [1, 2]. A standardized and optimal management of MPM is lacking, although detailed practical guidelines for the treatment of patients with MPM have been proposed [1–4]. Multimodality therapy consisting of chemotherapy, surgery and/or radiotherapy is centered on surgical resection in early stages. Currently, cisplatin is the drug of choice for the treatment of MPM, and carboplatin seems to have comparable efficacy [1, 3–7]. Combined with antifolates, both drugs are currently considered as the most effective regimen for MPM [7–10]. Nevertheless, cisplatin treatment results in a response rate of merely 14% and a median survival of less than seven months [8]. Carboplatin resulted in similar response rates ranging from 6 to 16% [8, 11].

Gender, histological subtype and hematological parameters have been identified as important prognostic parameters [5, 6]. Prognostic biomarkers related to survival of MPM patients and detectable in tumor specimens are however still lacking. Moreover, current guidelines clearly emphasize the need of innovative and novel therapies. For this, further basic research is needed to provide a more detailed insight into the pathogenesis and biology of MPM allowing opportunities for new treatment strategies [1].

Platin-analoga are genotoxic compounds inducing DNA damage [12] which in turn leads to TP53 induced cell cycle arrest and apoptosis [13]. It is however uncertain whether the failure of this mechanism alone is responsible for the impaired therapy response. Several studies have therefore attempted to identify molecular properties shared by MPMs to understand the basis of the poor treatment response [8, 11, 14–18].

Metallothionein (MT) is a family of cytoplasmic, cysteine-rich, low-molecular-mass proteins capable of binding heavy metals through the thiol groups of its cysteine residues [19]. There are four main isoforms expressed in humans (MT-I to MT–IV) with MT-I and MT-II representing the most prevalently expressed isoforms. Due to their role in various cellular processes, MTs have been studied in many malignancies [20]. MT has been shown to mediate resistance towards several toxic heavy metals, but compared to cadmium, the effect of MT on them is far less well studied [21]. We hypothesize that a possible mechanism of platinum containing chemotherapy failure may be based on enhanced MT expression by tumor cells.

## RESULTS

### Clinicopathological data

Of the 105 patients, 84 were males (80%) and 21 females (20%). The mean age at date of diagnosis was 65 years (median: 65, range: 34-82). Survival data of 100 patients were available for statistical analysis. 86 of the patients with available clinical data had succumbed to their disease, and 14 patients were still alive at the time of data collection. For five patients, no follow-up data were available. Median overall survival was 19.0 months (mean without censored patients: 23.9 months; 95% CI: 9.6-30.7 months, min: 1.2 months, max: 91.3 months), median progression-free survival was 7.5 months (mean without censored patients: 12.2 months; 95% CI: 5.9-12.3 months, min: 1.9 months, max: 64.7 months). 96 of the 105 cancers comprised MPM of the epithelioid subtype (91%), 5 of the biphasic subtype (5%), and 4 of the sarcomatoid subtype (4%). 70% of the patients initially underwent decortication, 20% pleurodesis, 5% pleuropneumectomy and for 5% patients the surgical therapy could not be assigned due to anonymization properties. The patients received platinum-based therapy (cis- or carboplatin) with varying numbers of chemotherapeutic cycles. With these therapeutic regimens, 42 patients showed stable disease (SD) (40%), 7 partial response (PR) (7%), and 54 progressive disease (PD) (51%) and for 2 patients the radiological response was not assessed (2%).

### Metallothionein expression in human MPM and associations with clinicopathological factors

**Figure 1:**
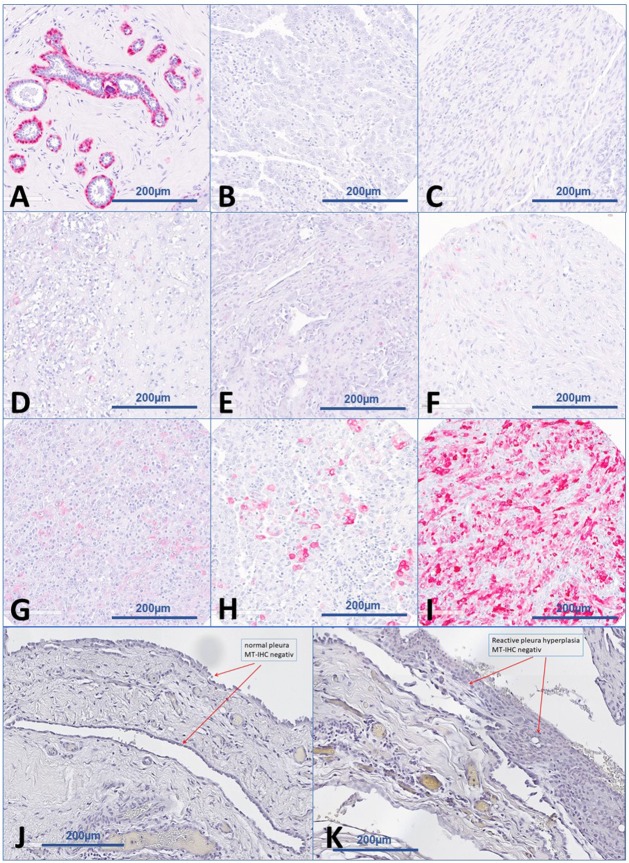
Examples of immunohistochemical staining scores **(A)** normal breast with myoepithelial cell as positive control. **(B)** immunohistochemically negative epitheloid MPM (Score 0). **(C)** Immunohistochemically negative sarcomatoid MPM (Score 0). **(D)** Immunohistochemically cytoplasmic positive MPM (Score 1). **(E)** Immunohistochemically nuclear positive MPM (Score 1). **(F)** Immunohistochemically cytoplasmic and nuclear positive MPM (Score 1). **(G)** Immunohistochemically cytoplasmic positive MPM (Score 2). **(H)** Immunohistochemically cytoplasmic and nuclear positive MPM (Score 2). **(I)** Immunohistochemically cytoplasmic and nuclear positive MPM (Score 3). **(J)** Immunohistochemically negative (Score 0) benign pleura. **(K)** Immunohistochemically negative (Score 0) reactive pleural hyperplasia.

**Figure 2:**
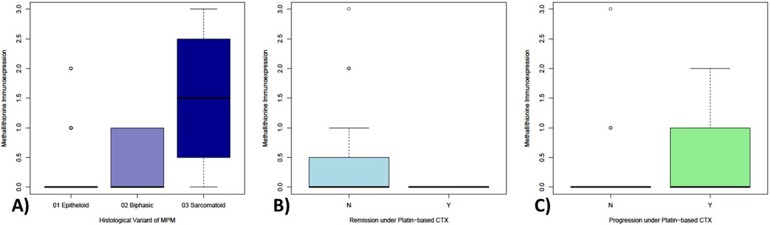
Differential expression of metallothionein in MPM samples with respect to histological and therapy-associated subgroups Metallothionein expression significantly increases from epitheliod (highlighted in light-blue) over biphasic (highlighted in middle-blue) up to sarcomatoid (highlighted in dark-blue) MPM **(2A)**. Epitheloid tumors show only MT-expression in a very limited population of MPM, whereas biphasic show an increased portion of expressing samples and sarcomatoid show MT-expression in the majority of samples. Strikingly, MT-immunoexpression could only be detected in tumors without remission **(2B)** and nearly exclusively on progressive tumors **(2C)** under platin-based chemotherapy.

21 tumors (20.0%) showed MT protein expression with 15 (71.4%) with Score 1; 5 (23.8%) with Score 2; and 1 (4.8%) with Score 3, respectively (Figure 1). Regarding the subcellular localization of MT-expression, forty-eight percent of the MT-positive tumors (n=10) showed a combined nuclear and cytoplasmic staining reaction, six tumors (28%) demonstrated a faint nuclear and 5 tumors (24%) a week cytoplasmic staining. MT-expression could rarely be detected in epithelioid variants of MPM, more often in biphasic tumors and in a gross of sarcomatoid ones (p-value: 0.0039; Figure 2A).

No significant association was found between MT expression and tumor progression (p=0.078) or remission (p=0.089) under platin-based chemotherapy. However, it is noteworthy that a positive staining reaction against MT (cut-off 1%) was observed only in progressive tumors (n = 21/54) with none of the remissive or stable (n = 0/51) showing any staining (Figure 2B/2C).

### Survival analyses

**Figure 3:**
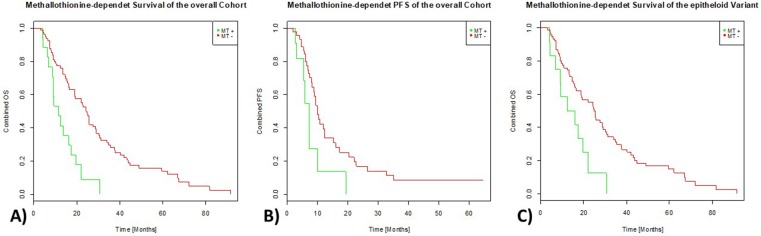
Effect of MT-immunoexpression on survival parameters MT expressing MPMs (green line) show a significantly shortened OS-rate **(3A)** as well as PFS rate **(3B)** compared to MPM samples without measurable expression of MT (red line). The same effect could be observed in epitheloid tumors only **(3C)**.

No association was observed between survival and patients’ gender (p=0.2643), but sarcomatoid and biphasic variants despite the small number of cases included showed significantly shortened patients’ overall survival (p=0.0036). With respect to functional scale-differences in biological systems, Cox-regression analyses were done in a linear and logarithmic scale revealing a significant association between expression of MT and shortened overall survival (OS) in a linear (p=0.0009) and logarithmic scale (p=0.0003; Figure 3A). For PFS, again a strongly reduced survival rate could be observed for MT expressing tumors (linear: p=0.0134, log: p=0.0152; Figure 3B). Whereas MT-negative MPMs showed a 3-years-overall-survival rate of approximately 35%, none of the 21 patients with positive metallothionein staining were still alive beyond this time period. Similarly, the metallothionein expressing tumors with the longest stable disease showed progression within 20 months of therapy, whereas 20% of MT-negative tumors were still stable or in remission at this time point.

Considering only tumors of the epitheloid histological subtype, Cox-regression analyses also revealed a significant association between expression of MT and shortened overall survival (OS) in a linear (p=0.0016) and logarithmic scale (p=0.0011; Figure 3C).

### Immunohistochemical staining of cell lines

All three analyzed MPM cell lines show detectable MT-immunoexpression levels, while the fibroblast cell line MCR-5 appears negative.

**Figure 4:**
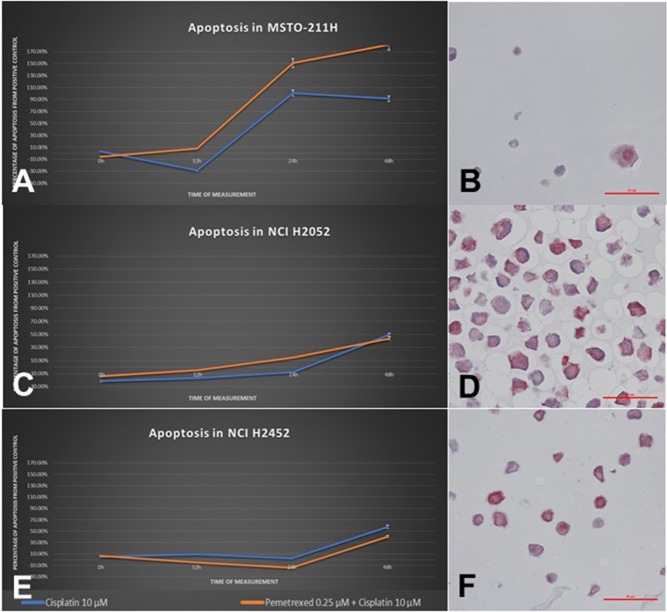
Cellular response of human MPM cell lines to treatment with cisplatin as a single agent and in combination with pemetrexed The left column **(A/C/E)** shows CASP3 activity of each cell line in a time-dependent manner, the right column **(B/D/F)** shows MT-immunostaining pattern of each respective cell line. MSTO-211H cells show a good induction of apoptosis due to cisplatin starting from 12h and a weak MT expression prominent in the nucleus. In contrast, the cell lines NCI-H2052 as well as NCI-H2452 show a significantly delayed and extenuated cellular response, reaching a CASP3-activity maximum of 30-40% at 48h. Both cell lines show a strong cytoplasmic protein expression of MT.

In principle, two different patterns of subcellular spatial distribution of MT protein expression could be observed pretreatment: 1) a weak MT expression prominent in the nucleus (MSTO-211H, Figure 4B) and 2) a strong and mainly cytoplasmic MT expression (NCI-H2452 and NCI-H2052, Figure 4D/4F).

**Figure 5:**
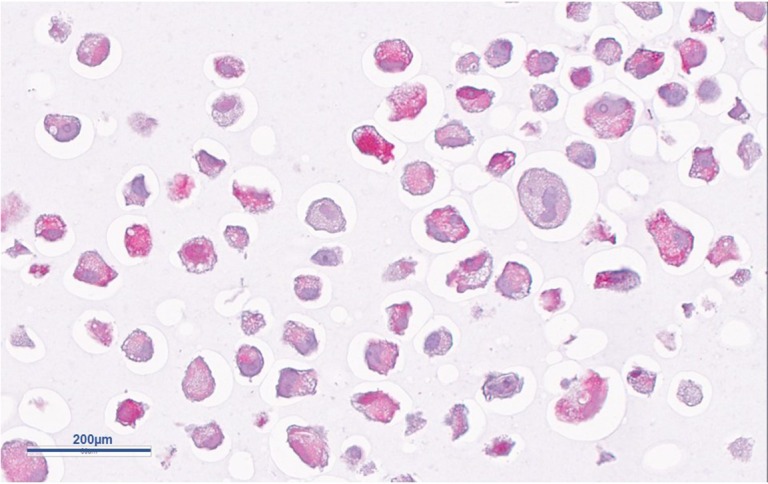
H&E staining of MSTO211H cells after 24h of incubation with cisplatin Nearly all cells show numerous regressive transformations like cytoplasmic vacuolization and fuzzy cell shape.

After 24h incubation with cisplatin, no significant changes in MT expression levels occurred in any of the cell lines, only MSTO-211H and MRC-5 histologically showed cytoplasmic vacuolization and overall regressive morphology (Figure 5). After 48h, NCI-H2052 and NCI-H2452 showed both an increasing staining intensity as well as an increased number of cells showing positivity for MTs (Table 1).

**Table 1:** Metallothionein immunoexpression in human MPM cell lines

	Cell type	Baseline^*^	24h^*^	48h^*^
**MRC-5**	benign	-	-	No vital cells
**MSTO-211H**	mesothelioma	(+)	(+)	No vital cells
**NCI-H2452**	mesothelioma	+	+	++
**NCI-H2052**	mesothelioma	++	++	+++

^*^ Time since incubation with cisplatin.

### Cell state analysis

While cell lines MRC-5 and MSTO-211H showed a strong induction of apoptosis and senescence by treatment with cisplatin, NCI-H2052 and NCI-H2452 cells showed a notably lower induction of apoptosis regarding cisplatin-treatment (Figure 4A/4C/4E). Also conspicuous was the delayed initial response, shifting from below 12h (MRC-5 and MSTO-211H) up to past 24h (NCI-H2052 and NCI-H2452).

No induction of necrosis could be observed with either of the used agents, except for the MSTO-211H cells (data not shown). Especially, wells treated with pemetrexed as single agent or in combination showed a necrotic effect.

### MT protein expression is associated with miRNA-566

**Figure 6:**
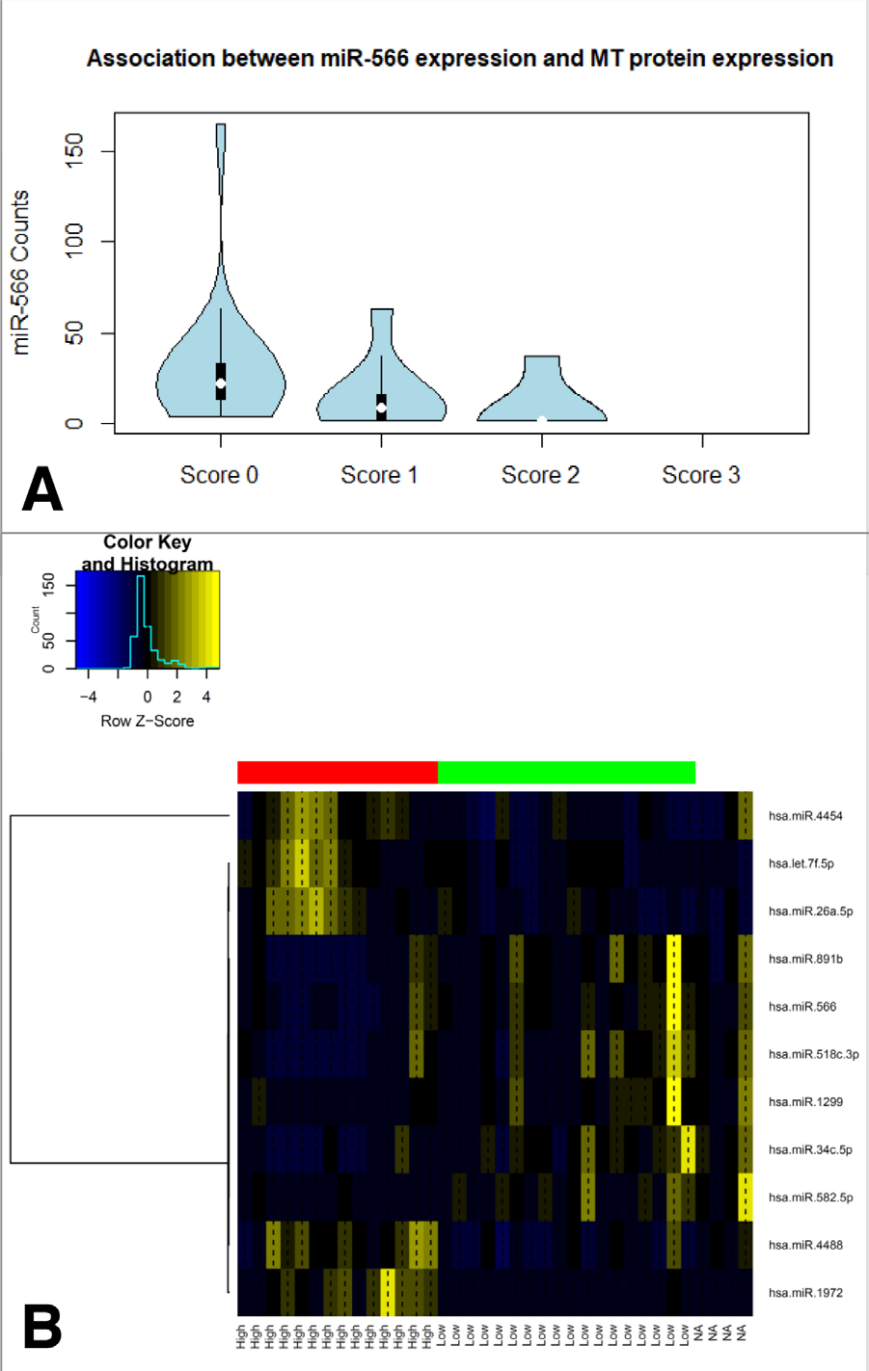
Associations between miRNA expression levels and MT proteinexpression in clinical MPM samples Overall, five miRNAs show an induction within the group of MT positive tumors and another six ones in MT negative samples **(B)**. Of special interest, as it probably directly regulates MT1A, MT2A as well as SLC31A1, miR566 expression clearly drops in samples showing detectable MT immunoexpression **(A)**.

Interestingly, a significantly altered miRNA expression signature could be identified in the subgroup of MT positive MPM compared to negative tumors. 34 miRNAs were shown to differ between both group, eleven of those got determined as highly significant (5 miRNAs are associated with high and 6 with a low MT-expression, Figure 6B). Of note, only a few of those miRNAs directly regulate metallothionein genes.

Strikingly, miR-566, probably regulating MT1A (binding probability: p=0.0203) as well as MT2A (binding probability: p=0.0203), is involved in the transport of platinum derived agents into the cell via direct regulation of the copper transporter SLC31A1. Moreover, increased miR-566 expression was significantly associated with higher MT protein levels (Figure 6A)

A GO-analysis revealed “cellular response to cisplatin” as well as “cellular response to radiation” both within the Top5 of influenced cellular processes (Table 2)

**Table 2:** GO-analysis results

GO category	p-value
**cellular response to cisplatin**	0.00004
**ion binding**	0.00199
**protein methyltransferase activity**	0.00212
**cell wall chitin metabolic process**	0.00247
**cellular response to radiation**	0.00247

### TCGA data sets and associations to promotor methylation levels

mRNA expression as well as methylation levels of none of the MT-family members showed a significant association to patients’ overall survival rate, except metallothionein 1D pseudogene (MT1DP, mRNA xp: p=0.0035, methylation: p=0.0270). Also, copy-number alterations (CNA) of none of the genes seem to be a relevant factor for overall survival time, although both deletions and especially gains could be detected (Supplementary Figure 2). Furthermore, no mutations in MT genes have been observed in any of the samples.

Interestingly, gene expression of nearly all MT family members appeared to be strongly dependent on DNA promotor hypomethylation, as only tumors with promotor methylation levels below approx.15% show gene expression levels at all (Supplementary Figure 2). CNA seem not to impact this. Furthermore, the different MT's seem to be co-regulated within each sample (Supplementary Figure 1).

## DISCUSSION

Due to their role in various cellular processes, MTs have been widely studied in various cancers [20]. MTs are known for their regulatory role during oxidative stress, their function involves ROS scavenging and ROS inhibition [22]. Furthermore, a depletion of NO-mediated cytotoxicity in MT overexpressing transgenic mice has been observed [23]. Metallothionein (MT) was reported to be a potential negative regulator of apoptosis, and various reports have suggested that it may play roles in carcinogenesis and drug resistance [24].

As described above, cisplatin shows cytotoxic effect depending on the concentration of heavy metal compounds. The heavy metal detoxifying effect from MT-I and MT-II may be the reason for the inactivation of alkylating drugs like platin-containing compounds (e.g. cis-platin or carbo-platin) [25, 26].

MTs are widely differently expressed in various tumor entities. While there is an overexpression in breast, uterine and skin carcinomas, they are downregulated in prostate carcinoma/adenoma, small cell lung carcinomas and hepatic carcinomas [27]. Whereas recent studies have showed a tumor suppressory role of some MT isoforms in papillary thyroid carcinoma, and cancers of the large intestine and melanomas [28–30], MTs have been shown to promote the progression of adenocarcinoma of the breast and lung [31–33]. Furthermore, the role of MTs in invasiveness, migration and sensitivity to anticancer agents has been widely studied [34–38].

A previous study showed a positive immunoreactivity against metallothionein in more than a half of diffuse MPM, and an increased MT protein expression level in patients with environmental asbestos exposure [39]. Additionally, in a portion of MPM samples, the MT locus was found to be silenced by DNA hypermethylation [40], resulting in a lack of MT expression and activity.

In our present study, immunohistochemical expression of MT was detected in approximately 20% of MPM samples, all in tumors with progressive clinical course despite chemotherapy. Therefore, our results indicate a possible resistance to platin-based chemotherapy in MPMs which exhibit enhanced MT-expression. Both, overall survival and progression-free survival are negatively correlated to detectable MT expression in MPM.

Similar discoveries have been already observed in ovarian, bladder, esophagus, liver and tongue cancer [41–43]. Furthermore, it is striking that MT expression could only be detected in progressive MPM but never in cancer showing remission under chemotherapy. However, in contrast to our data, no significant associations between MT expression in MPM and survival of the patients were found in another study [39].

Interestingly, the results rendered out of the TCGA data-base do not fully reflect our immunohistochemical findings, as mRNA expression levels of none of the MT genes was associated with overall survival. This could be explained in main by two reasons:

1. a dis-concordance between mRNA and protein expression levels have been described in many tissues like circulating monocytes [44], lung adenocarcinoma [45] or prostate cancer [46]; and
2. the survey of OS data is strongly dependent on the data collection and can differ with different parameters applied. Unfortunately, no data about response to therapy or progression-free survival are available within the TCGA data set.

Additional evidence for our hypothesis of MT-mediated platin-resistance in MPM could be generated by our *in vitro* inhibitory experiments. The direct association between strong cytoplasmic staining, clearly reduced apoptosis as well as cellular senescence rate completes the picture of clearly shortened OS and PFS and early therapy failure under platin treatment. Of note, contrary to the patient samples, all investigated cell lines showed at least a weak nuclear MT staining. We explain this by the difference in formalin-fixation between tissue and cell blocks, leading to a shift in the detection limit and staining background.

Findings reveal that subcellular localization of MT is important for its predictive and prognostic value [42, 43]. In ovarian cancer, nuclear MT-I expression was shown to be induced during platin-based treatment which led to cancer progression, relapse and increased mortality, whereas no relationship was found between cytoplasmic expression and patients’ outcome, possibly by inhibiting the binding of cisplatin to DNA. In our study on mesothelioma biopsies obtained before treatment, the majority of strong and medium MT-expressing tumors showed a mixed cytoplasmic and nuclear staining. Pure and faint nuclear or cytoplasmic reactions were found in scattered tumor cells within the group of low-MT-expressers.

*In vivo* experiments show an additional resistance to a broad range of anticancer drugs as bleomycin, melphalan, ara-c, cytarabine, etoposide, doxorubicin methotrexate as well as radiotherapy [20, 27, 38, 47–49]. However, this association is discussed controversially, making MT expression to be a non-reliable marker for prognosis [43]. Eid et al. [50], Meijer et al. [51], and Endo et al. [52] predicted a better response to cisplatin.

Resistance against alkylating drugs is one of the main causes of therapeutic failure in highly aggressive human carcinomas. Two main mechanisms of the possible role of MTs are widely discussed in the literature. Firstly, MTs are capable to bind platin-based chemotherapeutic agents as well as their metabolites directly, thereby preventing them from inducing DNA damage [20, 53–55]. Especially the mechanism of binding, inactivation and sequestering of platin has been clarified. The structural studies revealed that MTs bind seven Zn(II) or Cd(II) through cysteine thiolates forming two metal–thiolate clusters in the β-domain as well as the α-domain of the protein [56, 57]. As the affinity of Pt(II) for cysteine thiolates exceeds that of Zn(II) for 10^7^ fold, Zn(II) is substituted by Pt(II) [57, 58]. However, recently it became evident that the extent, to which MT sequesters platinum drugs, depends not only on the type of cancer, but also on the interaction of MT with the ligand sphere of the platinum center [59].

Secondly, MTs might act as a negative regulator of the effect of the chemotherapy by sequestering zinc ions [60, 61], thereby regulating proteins based on zinc ions as cofactor like p53. We also compared MT expression presented within this study with results on p53 and mdm2 from previous investigation from our group [16, 18, 62–64]. However, we could not define significant associations between the two pathways.

The anti-MT antibody (clone E9) we used is able to detect both MT1 and MT2 isoforms [71], of which MT2 is inducible by heavy metals and capable of binding them. Our results support the hypothesis that in MPM it is rather the heavy-metal-binding activity of MT2, which might be causally related to the resistance to platin-based chemotherapy.

Notwithstanding this, p53 is an essential regulator for apoptosis in case of DNA damage repair and recognition [60] and plays an important role in tumorigenesis and response to alkylating chemotherapeutics [16, 18, 62–64]. Previously, possible correlations to clinic-pathological data with the tumor suppressors p53 and MTs were analyzed in MPM samples but revealed no statistical association as well as no correlation to each other [39]. Nevertheless, in previous experiments, we found a significantly poorer survival in patients with MDM2-expressing MPMs of the epithelioid and biphasic subtypes [62–64]. This might be caused by MDM2-mediated inactivation of functional p53 protein in the setting of *TP53* wild-type. Taken together both mechanisms, MDM2 and MT expression might provide an explanation for the way therapy resistance in MPMs is acquired.

As one possible translational strategy based on these results, a supplementation of zinc may overcome the issue of MT-driven platin-resistance by saturation of all free Pt(II) binding sites with high concentrations of Zn(II). Kocdor et al. could show *in vitro*, that the supplementation of 50μM zinc into the cell culture media reduces the IC50 value of docetaxel from 20.4μM to 8.1, the supplementation of 100μM zinc even down to 6.6μM [65]. Contrarily, Alscher et al. could show an induction of MT gene- and protein expression levels in mesothelial cells via zinc [66]. This will reduce the sensitivity to chemotherapy, and it remains to be proven if there is a protective effect of this to either benign pleural tissue or pleural mesothelioma or not.

## MATERIALS AND METHODS

### Demographic data

Formalin-fixed paraffin-embedded (FFPE) tumor specimens from 105 patients suffering from MPM were screened. Tumor classification was based on the respective WHO classification of tumors (2004) [67], and the TNM-staging on the UICC classification [68]. Both were confirmed by two experienced pathologists (JW, TM). The study included only patients with MPM, treated at the West German Cancer Centre or the West German Lung Centre (Essen) between 2006 and 2009 and the Helios Klinikum Emil von Behring (Berlin) between 2002 and 2009. Inclusion criteria were the availability of both sufficient tumor material and a complete set of data related follow-up and treatment.

Furthermore, gene expression data (RNA Seq) of 87 malignant pleural mesothelioma patients were retrieved from the The Cancer Genome Atlas (TCGA) database (courtesy of National Cancer Institute, National Human Genome Research institute, Bethesda, MD, US).

### Clinicopathological data

Most of the tumors showed epithelioid histology, and only a few cases of biphasic (n = 5) and sarcomatoid (n = 4) MPM were available for the study. All specimens were collected prior to systemic treatment. Study surveillance was terminated on August 31, 2014. Patients’ clinic-pathological and clinical data are summarized in Table 3.

**Table 3:** Overview of patients characteristics

Number of patients	105
Gender	
male	84
female	21
unknown Gender	0
Histological subtype	
epitheliod	96
biphasic	5
sarcomatoid	4
Age	
Mean | Median age at diagnosis (months)	65 | 65
Range (months)	34-82
OS	
Deceased	86
Alive	14
Lost-of-FU	5
Median | Mean OS (months)	19.0 | 23.9
95% CI	9.6-30.7
PFS	
Partial remission (initial)	7
Stable disease (initial)	42
Progressive disease (initial)	54
Unknown response	2
Median | Mean PFS (months)	7.5 | 12.2
95% CI	5.9-12.3

Response data were evaluated centralized using the modified Response Evaluation Criteria in Solid Tumors (modRECIST) for assessment of radiological response in MPM [69]. As described elsewhere, for a better stratification, remission was defined by complete response (CR) and partial response (PR) versus stable disease (SD) and progressive disease (PD) [70]. Likewise, progression was defined by CR and PR and SD versus PD [70].

The study was conducted retrospectively and was approved by the Ethics Committee of the Medical Faculty of the University Duisburg-Essen (identifier: 14-5775-BO). The investigation conforms to the principles outlined in the declaration of Helsinki.

### Cell line experiments

For *in vitro* experiments, the cell lines MRC-5, MSTO-211H, NCI-H2052 and NCI-H2452 were used. Human MPM cell lines were obtained from the American Type Culture Collection in 2012-08 (Manassas, VA, USA). The cell lines were authenticated and tested for contaminations by using a commercial service (Multiplexion, Heidelberg, Germany) and were last re-tested directly after the experiments were finished.

Based on reviewing the literature, concentrations for the cytostatic drugs were estimated [71–75].

### Maintenance of eukaryotic cells

Cultivation of cells was done at standard incubator conditions (humidified atmosphere, 5% CO2, 37°C). Cells were maintained in T-75 or T-175 cell culture flasks (Greiner Bio-One, Kremsmünster, Austria) using Roswell Park Memorial Institute (RPMI) -1640 medium plus 10% fetal calf serum (FCS) and 1% Penicillin and Streptomycin (P/S, Thermo Fisher Scientific, Massachusetts, USA) for tumor cell lines. For MRC-5 cells, minimal essential medium (MEM) plus 10% FCS and 1% P/S was used. Cells were grown until 95-100% confluency. Subsequently, after washing twice with 5 ml Dulbecco's Phosphate Buffered Saline (DPBS, Thermo Fisher Scientific) cells were trypsinized with 2 ml of 0.05% Trypsin-EDTA (Thermo Fisher Scientific) for 5-10 min at 37°C. Trypsination was inactivated by resuspending the cells in 8 ml medium for passaging or cell counting. For cell counting, 10 μl of cell suspension was mixed with 10 μl of trypan blue (Thermo Fisher Scientific) and 10 μl of the mix were transferred to the hemocytometer (Brand, Wertheim, Germany).

### Treatment of MPM cell lines with cytostatic agents cisplatin and pemetrexed

The cell lines were analyzed for apoptosis, senescence and necrosis during treatment with pemetrexed and/or cisplatin [76]. These agents was supplied by Selleckchem (Cisplatin: S1166, Pemetrexed: S1135, Houston, USA). Cisplatin was solubilized by Dimethyl sulfoxide (DMSO, Sigma-Aldrich, Missouri, USA) and pemetrexed was solubilized by dH2O.

For the treatment of cells, 5,000 cells (50 μl) were seeded into microplates 96/U (Eppendorf, Hamburg, Germany) that are suitable for luminescence detection. The cells could attach overnight at 37°C and 5% CO2. At the next day, 50 μl of fresh medium either containing one of the treatment agents or without additive was applied to each well. The concentrations of the agents were 0.25 μM for pemetrexed and 10 μM for cisplatin. Cisplatin was combined with pemetrexed, representing the approved chemotherapeutics for MPM.

### Cell state analysis

Senescence of the cells was analyzed by a luminescent-based activity assay of living cells. The assay was performed using the CellTiter-Glo® Luminescent Cell Viability Assay kit (G7571, Promega). The CellTiter-Glo® reagent was prepared according to the protocol provided by the manufacturer. Digitonin (30 μg/ml, supplied by the kit) served as positive control to measure a decrease of cellular viability of 100%. 10 μl of digitonin was given to the cells 15 min before cell lysis. Cell lysis was induced by applying 100 μl of CellTiter-Glo® reagent to each well and incubated for 10 min of at room temperature.

The necrosis of cells was analyzed using the CytoTox-Glo® Assay kit (G9191, Promega). 5,000 cells per reaction were used. 100 μl of required cells/well were placed in a white 96-well plate. The AAF-Glo® reagent was prepared according to the protocol provided by the manufacturer. Ionomycin (S1160, Selleckchem) was used as positive control. Two hours before measurement, 50 μl of Ionomycin (100 μM), was added to the cells. After adding 50 μl of the AAF-Glo® reagent to each well, cells were incubated for 15 min at room temperature, protected from light.

The apoptotic potential of the cells was analyzed using the Caspase-Glo® 3/7 Assay (G8093, Promega). 5,000 cells per reaction were used. 100 μl of required cells/well were placed into a white 96-well plate. The Caspase-Glo® reagent was prepared according to the protocol provided by the manufacturer. Staurosporine (10 μM, S1421, Selleckchem) served as positive control and was given to the cells 4 h before measurement. After adding 100 μl of Caspase-Glo® reagent to each well, cells were incubated for 30 min at room temperature.

All reactions were measured using a luminometer (Glo Max Multi + Detection System; Promega).

### Immunohistochemistry

Formalin-fixed tissues were embedded in paraffin and processed into 4μm thick slides for histomorphological diagnosis (H&E sections) and immunohistochemistry (IHC). For systematic immunohistochemical investigations, tissue microarrays (TMA) were constructed from paraffin blocks. Three cores with a diameter of 0.6 mm were taken from different areas of each tumor to take possible tumor heterogeneity into account. When feasible, a core containing only normal lung tissue and unaffected pleura was taken from every specimen for negative control purposes. Immunohistochemistry was performed according to standard protocols using an automated staining device (Ventana Discovery XT, Munich, Germany). After validation on reference tissues (myoepithelial cells of normal breast tissue) and normal pleura samples as a negative control, the immunohistochemical investigations were performed with a monoclonal primary antibody (clone E9) (Dako/Agilent, Santa Clara, CA, US) directed against at an epitope shared by MT-1and MT-2 [77]. Pre-treatment for antigen retrieval was performed by heating in citrate buffer (Ultra Cell Conditioning Solution II, Ventana Medical Systems, Basel, CH) at pH 6, 90°C for 30 minutes.

Immunohistochemical MT protein expression was evaluated by a senior pathologist and a trained scientist using a consultation light microscope (Nikon Eclipse 80i, Nikon Ltd. Dusseldorf, Germany). A semi-quantitative four-tier IHC scoring system was used based on the percentage of tumor cells with a positive nuclear and/or cytoplasmic immunoreaction with the antibody directed against metallothionein irrespective of staining intensity (Score 0: no immunohistochemical signal; Score 1 (weak expression): 1-5% MT-positive tumor cells; Score 2 (moderate expression): 6-50% MT-positive tumor cells; Score 3 (strong expression): >50% MT-positive tumor cells) [77, 78].

### RNA extraction and RNA integrity assessment

According to the manufacturer's recommendations, three to five paraffin sections with a thickness of 4μm per sample were deparaffinized with xylene prior to total RNA extraction including small RNAs using the miRNeasy FFPE kit (Qiagen, Hilden, Germany). RNA concentration was measured using a Qubit 2.0 fluorometer (Life Technologies) appertaining the RNA broad-range assay. RNA integrity was assessed using a Fragment Analyzer (Advanced Analytical Inc., Ames, IA, USA) appertaining DNF-489 standard sensitivity RNA analysis kit.

### NanoString miRNA codeset design and expression quantification

The commercially available nCounter miRNA Expression Assay v2.1 (NanoString Technologies, Seattle, WA, USA) containing probes and miRTags for the 800 most important known miRNAs described in the context of cancerogenic events was chosen for miRNA Expression analysis. Five potential reference genes (ACTB, B2M, GAPDH, RPL19, RPLP0) were also included in the CodeSet for biological normalization purposes. Probe sets and miRTags for each target in the CodeSet were designed and synthesized at NanoString Technologies (Seattle, WA, USA). 100 ng total RNA were analyzed for each sample (in a final volume of 3 μl). The sample preparation in the nCounter Prep Station (NanoString) was carried out by using the high-sensitivity protocol (3h preparation). The cartridges were measured at 555 fields of view in the nCounter Digital Analyzer (NanoString).

### NanoString data processing and statistical analysis

Both the statistical and graphical analyses were performed with the R statistical programming environment (v3.4.2).

NanoString data processing was done using the *NanoStringNorm* [79] and the *NAPPA* package, respectively. Considering the counts obtained for positive control probe sets raw NanoString counts for each gene were subjected to a technical factorial normalization, carried out by subtracting the mean counts plus two-times standard deviation from the CodeSet inherent negative controls. Afterwards, a biological normalization using the geometric mean of the 100 top expressed miRNAs was performed. Additionally, all counts with p>0.05 after one-sided t-test versus negative controls plus 2x standard deviations were interpreted as not expressed to overcome basal noise.

Before starting the analysis, the Shapiro-Wilks-test was applied to test for normal distribution of each data set. Based on the results, either a parametric or non-parametric test was applied. The exact Wilcoxon Mann-Whitney Rank Sum test was used to test associations between the mean protein expression obtained from three cores and dichotomous variables (gender).

Overall survival was calculated by producing single-factorial and combined Kaplan-Meier curves. Survival analysis was done by Cox-regression (COXPH-model), and statistical significance was determined using likelihood ratio test, Wald test and Score (logrank) test. Kaplan-Meier curves with a confidence interval of 95% (95% CI) were calculated based on existing survival data and combined survival curves were performed.

The level of statistical significance was defined as p<0.05.

## CONCLUSION

Our results show that immunohistochemical MT expression is significantly correlated with poor overall and progression-free survival in MPMs under platin-based chemotherapy, thereby suggesting its possible role as a predictor of resistance to platin-based chemotherapy.

## SUPPLEMENTARY MATERIALS FIGURES


