# Supplementary material for: Immunohistochemically detectable metallothionein expression in malignant pleural mesotheliomas is strongly associated with early failure to platin-based chemotherapy
Source: Oncotarget. 2018 Apr 27;9(32):22254–68. doi: 10.18632/oncotarget.24962 (PMC5976462; doi:10.18632/oncotarget.24962)
Supplement: Supplementary file 1 [file oncotarget-09-22254-s001.pdf]

Immunohistochemically detectable metallothionein expression in malignant pleural mesotheliomas is strongly associated with early failure to platin-based chemotherapy

SUPPLEMENTARY MATERIALS

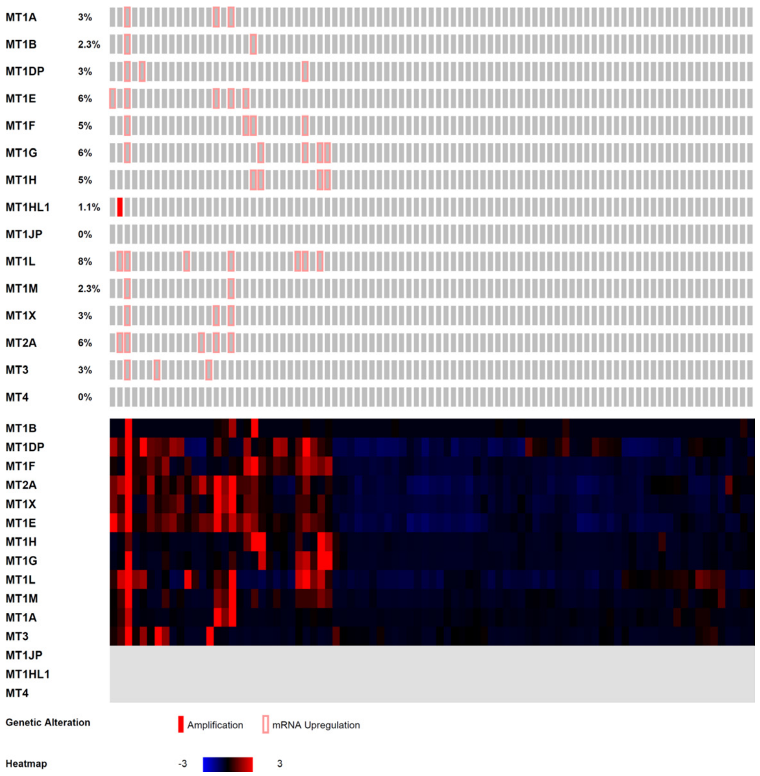

Supplementary Figure 1: Overview of alteration-distribution in different metallothionein genes over all patients. Mostly, there seems to be a co-regulation of different MT genes within each affected sample.

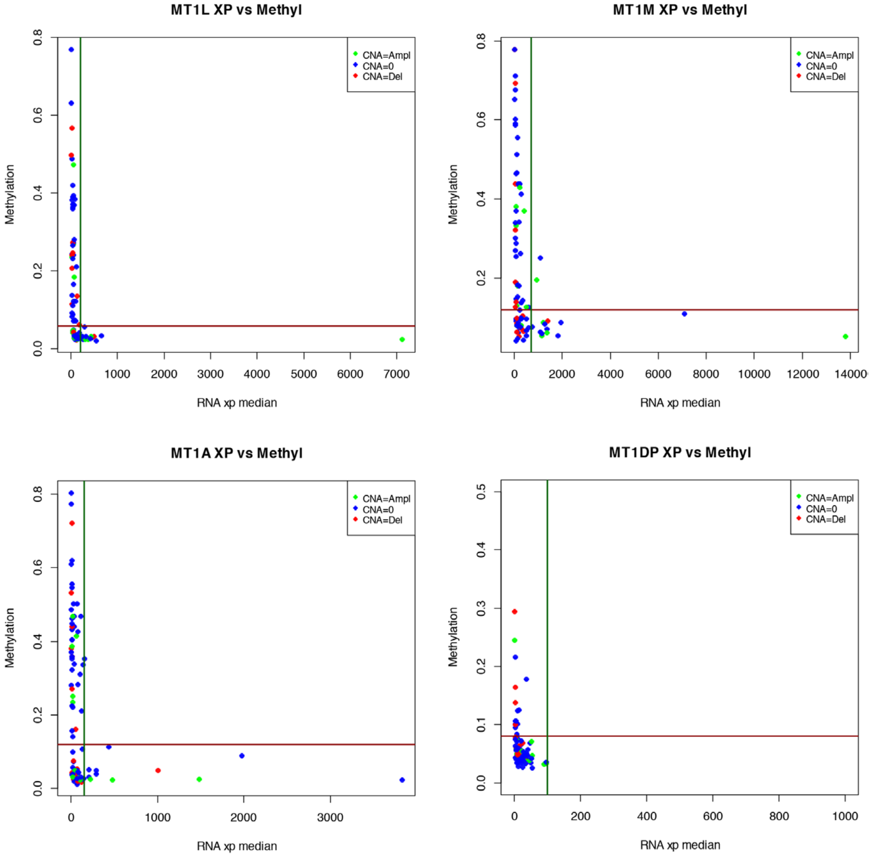

Supplementary Figure 2: Exemplary associations between mRNA expression levels (x-axis) and promotor methylation (y-axis) of MT genes. Dot-colors indicate CNA status of each sample spotted.
